# Variation of chemical composition of essential oils in wild populations of *Thymus algeriensis *Boiss. et Reut., a North African endemic Species

**DOI:** 10.1186/1476-511X-11-28

**Published:** 2012-02-20

**Authors:** Nacim Zouari, Imen Ayadi, Nahed Fakhfakh, Ahmed Rebai, Sami Zouari

**Affiliations:** 1 Laboratoire de Biochimie et de Génie Enzymatique des Lipases, Ecole Nationale d'Ingénieurs de Sfax, BP 1173, 3038 Sfax, Tunisia; 2 Laboratoire de Microorganismes et de Biomolécules, Equipe de criblage moléculaire et cellulaire, Centre de Biotechnologie de Sfax, BP 1177, 3018 Sfax, Tunisia; 3 Laboratoire de Génie Enzymatique et de Microbiologie, Ecole Nationale d'Ingénieurs de Sfax (ENIS), BP 1173, 3038 Sfax, Tunisia; 4 Laboratoire d'Ecologie Pastorale, Institut des Régions Arides, 4119 Médenine, Tunisia

**Keywords:** *Thymus algeriensis*, Biodiversity, Essential oil, Chemical composition, Discriminant analysis

## Abstract

**Background:**

*Thymus algeriensis *is an endemic aromatic plant to Tunisia largely used in folk medicine and as a culinary herb. The bulks aromatic plants come from wild populations whose essential oils compositions as well as their biological properties are severely affected by the geographical location and the phase of the plant development. Therefore, the aim of the present work is to provide more information on the variation of essential oil composition of *T. algeriensis *collected during the vegetative and the flowering phases and from eight different geographical regions. Besides, influence of population location and phenological stage on yield and metal chelating activity of essential oils is also assessed.

**Methods:**

The essential oil composition of *Thymus algeriensis *was determined mainly by GC/FID and GC/MS. The chemical differentiation among populations performed on all compounds was assessed by linear discriminate analysis and cluster analysis based on Euclidean distance.

**Results:**

A total of 71 compounds, representing 88.99 to 99.76% of the total oil, were identified. A significant effect of the population location on the chemical composition variability of *T. algeriensis *oil was observed. Only 18 out of 71 compounds showed a statistically significant variation among population locations and phenological stages. Chemical differentiation among populations was high. Minor compounds play an important role to distinguish between chemical groups. Five chemotypes according to the major compounds have been distinguished. Chemotypes distribution is linked to the population location and not to bioclimate, indicating that local selective environmental factors acted on the chemotype diversity.

**Conclusions:**

The major compounds at the species level were α-pinene (7.41-13.94%), 1,8-cineole (7.55-22.07%), *cis*-sabinene hydrate (0.10-12.95%), camphor (6.8-19.93%), 4-terpineol (1.55-11.86%), terpenyl acetate (0-14.92%) and viridiflorol (0-11.49%). Based on major compounds, the populations were represented by (α-pinene/1,8-cineole/*cis*-sabinene hydrate/camphor/viridiflorol), (1,8-cineole/camphor/terpenyl acetate), (α-pinene/1,8-cineole/camphor), (1,8-cineole/camphor/4-terpineol) and (α-pinene/1,8-cineole/*cis*-sabinene hydrate/camphor/4-terpineol) chemotypes. Variation of phenological stage did not have a statistically significant effect on the yield and metal chelating activity of the essential oil. These results can be used to investigate the geographical location and the harvesting time of this plant for relevant industries.

## Background

In the last few years, there has been an increasing concern regarding the safety and potentially adverse effects of synthetic chemicals used for food preservation or in medicine. Therefore, the commercial development of medicinal plants as new sources of bioactive products to enhance human health and food preservation is of prime importance. Essential oils extracted by hydrodistillation from aromatic plants are needed for their various biological and pharmacological properties. However, several factors, namely climatic, geographic conditions and growth stage of collected plants may severely affect essential oil yield, their composition and their biological properties. Thus, studies of chemical variability of essential oil in relation to environmental factors might provide information on what determines its chemical polymorphism. In addition, knowledge of the chemical composition of essential oils is a very important quality criterion for their marketing and contributes to their valorization.

*Thymus *(Lamiaceae) is a large genus divided in eight sections, comprising more than 250 species particularly prevalent in the Mediterranean area. *Thymus algeriensis *Boiss. et Reut., which is endemic to Tunisia and Algeria, is an herbaceous fragrant plant largely used, fresh or dried, as a culinary herb [1]. Furthermore, this plant is also widely used in folk medicine against illnesses of the digestive tube and antiabortion [2]. Recently, the *T. algeriensis *essential oil was found to possess an interesting inhibitory activity towards angiotensin I-converting enzyme suggesting the potential of this plant as an antihypertensive agent [3]. In Tunisia, *T. algeriensis *populations are distributed from the sub-humid to the lower arid bioclimates at altitudes ranging from 120 to 1100 m. The species grows on poor fertile calcareous soils and occurs in scattered and small populations. *T. algeriensis *is a short lived, diploid (2n = 2x = 30) and gynodioecious shrub. It reproduces by seeds and can reach 20-50 cm in height. The leaves are opposite and linear/lanceolate (6-12 mm). The flowers, with ovate bracts and pink purplish or whitish purple corolla, are small (5-7 mm). Flowering takes place between April and June.

Previous works on *T. algeriensis *showed important intraspecific chemical variability of the essential oils among samples according to the geographical regions [3-6]. However, there are no researches in assessment of essential oil variations at the vegetative stage of this plant and in different geographical locations. Therefore, the aim of the present work is to provide more information on the variation of volatiles of Tunisian *T. algeriensis *collected during the vegetative and the flowering phases and from eight different localities and to determine in which way this would affect the corresponding oils yields and their metal chelating activities.

## Results and discussion

### Identified essential oil compounds

**Figure 1:**
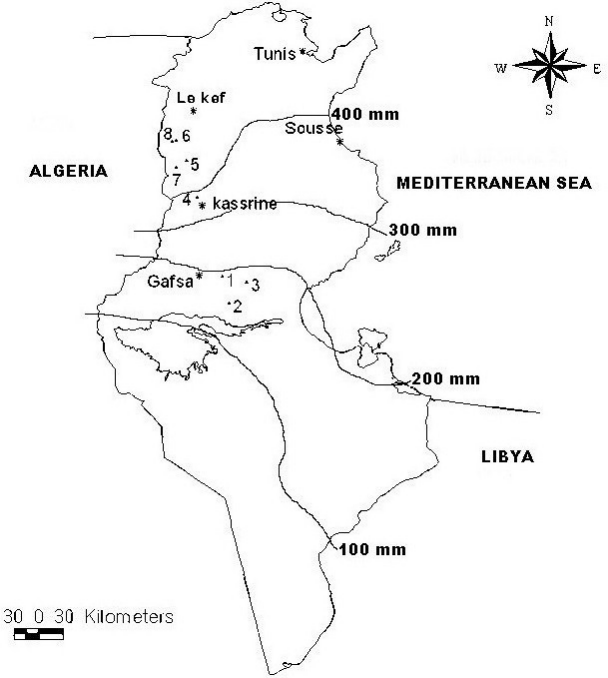
**Goegraphical localization of the 8 sites of Tunisian *T. algeriensis *populations**. For the detailed description of the locations: see Table 1.

Eight wild populations of *T. algeriensis *from different regions were collected during the vegetative (S1) and the flowering (S2) stages. They belonged to 3 bioclimatic zones. Populations 1, 2 and 3 were located at the South West of Tunisia (Gafsa region) with an inferior arid climate characterized by a mean rainfall of 100-200 mm/year, while populations 5, 6, 7 and 8 were localized at the North West of Tunisia (Le Kef region) characterized by a superior semi-arid climate (rainfall: 400-500 mm/year). The intermediate population 4 was from the inferior semi-arid bioclimate and characterized by a mean rainfall of 300-400 mm/year. The altitudes ranged from 192 m (population 3) to 800 m (population 7) (Table 1 and Figure 1).

**Table 1:** Location and main ecological factors of the 8 *T. algeriensis *populations analyzed

No^a^	Locality	Bioclimatic zone	Rainfull (mm/year)	Latitude	Longitude	Altitude (m)
1	Zannouch	Inferior arid	100-200	34° 24' 43" N	009° 04' 26" E	536
2	Oued Om Ali	Inferior arid	100-200	34° 07' 44" N	009° 09' 54" E	265
3	Ayaycha	Inferior arid	100-200	37° 21' 05" N	009° 23' 32" E	192
4	Sidi Harrath	Inferior semi-arid	300-400	35° 14' 55" N	008° 45' 09" E	667
5	Dachra	Superior semi-arid	400-500	35° 38' 31" N	008° 36' 35" E	693
6	Djebel Slata	Superior semi-arid	400-500	35° 51' 32" N	008° 28' 27" E	670
7	Haydra	Superior semi-arid	400-500	35° 34' 16" N	008° 28' 20" E	800
8	Kalaat Senan	Superior semi-arid	400-500	35° 51' 02" N	008° 25' 09" E	541

^a^The numbering refers to the *T. algeriensis *populations.

The chemical composition of all the oil samples was mainly investigated using both GC/FID and GC/MS techniques. The percentages and the retention indices of the identified compounds of these essential oils were listed in Table 2 in the order of their elution on the HP-5MS column. Seventy-one compounds, representing 88.99 to 99.76% of the total essential oil, were identified and separated on the basis of their chemical structures into 5 classes (Table 2). Whatever the phenological stage, all these essential oils were characterized by very high percentage of monoterpenes (49.91-90.33%) and especially the oxygenated ones (32.01-62.18%) which constituted the predominant class as was found previously for *T. algeriensis *[3,5,6]. The sesquiterpenes were also represented mainly by oxygenated sesquiterpenes (2.92-21.84%) in contrast to what has been observed by Ben El Hadj Ali et al. [6] where the amount of oxygenated sesquiterpenes did not exceed 4.6% of the total essential oil of *T. algeriensis*. Although all the studied samples could be classified as oxygenated monoterpene-rich oils, they have shown wide range of variations in their compounds. The essential oils chemotypes detected in Tunisian *T. algeriensis *populations based on major compounds as well as the clusters among populations based on all the oil compounds are shown below.

**Table 2:** Mean percentage of compounds (%) in essential oils of the 8 *T. algeriensis *populations during the vegetative (S1) and the flowering (S2) stages

			I. Aride^c^						I. Semi-Aride^c^		S. Semi-Aride^c^								*P1 *Fisher test	*P2 *Friedman test	*P3 *Kruskal-Wallis test
									
No^a^	Compounds	RI^b^	1		2		3		4		5		6		7		8				
			S1	S2	S1	S2	S1	S2	S1	S2	S1	S2	S1	S2	S1	S2	S1	S2			
1	Tricyclene	924	0.27	0.26	0.19	0.21	0.17	0.31	0.33	0.11	0.48	0.24	0.47	0.40	0.31	0.34	0.34	0.2	**	0.464	**
2	α-Thujene	929	0.41	0.27	0.85	0.71	0.33	0.3	0.14	0.14	0.17	0.33	0.25	0.29	0.21	0.29	0.15	0.2	***	0.127	**
**3**	α**-Pinene^d^**	936	**10.49**	**9.68**	**7.41**	**9.80**	**8.97**	5.60	6.58	0.98	**9.18**	**13.25**	**11.74**	**10.34**	**13.44**	6.38	**13.94**	**12.4**	***	0.170	**
4	Camphene	951	3.84	3.89	3.22	3.51	3.48	4.16	4.11	2.22	5.59	4.01	6.06	5.43	5.58	4.38	6.35	4.94	***	0.106	***
5	Verbenene	956	0.29	0.40	0.03	0.10	0.10	0.35	0.72	0.18	0.66	0.37	0.65	0.60	0.46	0.38	0.43	0.44	***	0.304	**
6	Sabinene^e^	975	3.37	2.12	3.15	4.40	2.90	0.96	0.66	0.76	0.67	0.92	0.73	0.94	0.98	0.93	0.70	0.93	***	0.060**†**	***
7	β-Pinene	978	2.78	2.13	4.03	4.29	2.86	1.47	1.65	1.73	1.96	3.40	2.72	3.00	2.37	1.87	2.41	3.22	***	0.318	**
8	β-Myrcene	992	0.82	0.29	0.84	0.78	0.44		1.58	0.46	0.64	0.60	0.64	0.74	0.65	0.50	0.38	0.67	ns	0.429	ns
9	α-Phellandrene	1006	0.19	0.16	0.11	0.09	0.07	0.18	0.18		0.16	0.21	0.18	0.18	0.14	0.12	0.11	0.04	*	0.398	*
10	α-Terpinene	1018	1.13	1.18	1.65	0.89	1.52	3.48	0.44		0.45	0.27	0.50	0.51	0.47	2.24	0.32	0.23	***	0.095**†**	***
11	p-Cymene	1027	1.78	1.81	1.20	1.80	1.73	4.2	1.74	1.65	1.98	1.13	1.53	1.23	1.29	3.68	0.75	1.10	**	0.363	*
12	Limonene	1030		0.32	0.28	0.71	1.00	0.7					0.35		1.16	0.21	1.06	0.54	na	0.222	*
**13**	**1,8-Cineole^d^**	1035	**10.91**	**15.79**	**7.55**	**8.73**	**9.00**	**10.87**	**18.02**	**13.82**	**14.44**	**14.73**	**17.90**	**18.46**	**22.07**	**12.45**	**20.48**	**15.36**	***	0.230	***
14	*trans*-β-Ocimene	1049	1.55	0.57	0.87	0.08	0.86	0.21	0.35	0.16	0.26	0.94	0.70	0.39	0.83	0.31	0.15	0.30	ns	0.540	ns
15	γ-Terpinene	1060	1.81	1.98	3.15	1.68	2.65	5.42	1.00	0.10	0.82	0.53	0.88	0.85	0.81	3.63	0.53	0.56	***	0.230	***
**16**	***cis*-Sabinene hydrate^d, e^**	1070	2.83	0.88	**9.86**	2.59	**12.95**	2.79	0.10	0.15	1.22	0.54	0.66	0.76	1.08	1.85	0.98	1.06	***	0.095**†**	***
17	*cis*-Linalool oxide	1074	0.30	0.37		0.05		0.31			0.25	0.12	0.26	0.28	0.17	0.28	0.20	0.09	***	0.152	**
18	Camphenilone	1086	0.15	0.08		0.08	0.05	0.32	0.31	0.40	0.52	0.44	0.35	0.38		0.31			***	0.078**†**	***
19	Terpinolene	1089	0.98	1.21	0.79	0.55	0.97	1.94	0.51		1.25	0.52	0.61	0.94	0.52	1.37	0.53	0.42	***	0.241	**
20	Linalool	1095	2.95	2.69	0.34	0.54	2.16		0.79	1.07	1.69	2.20	2.07	2.20	1.57	0.44	1.77	2.42	**	0.207	**
21	*trans*-Sabinene hydrate	1101	0.67		1.45	0.64	1.17	1.73								1.09			na	0.151	*
22	p-Menth-2-en-1-ol	1119	0.11	0.41	0.60	0.27	0.60	1.07	0.43	0.36			0.08		0.29	0.71		0.16	***	0.107	***
23	Campholenal	1122	1.41	1.02		0.40	0.70	0.88	1.31	0.71	2.76	1.35	1.82	1.91	1.41	1.03	1.44	1.66	***	0.065**†**	***
24	Nopinone	1135	0.15	0.21		0.29	0.13	0.25	0.50		0.48	0.14	0.33	0.32	0.30	0.24	0.29	0.15	ns	0.885	*
25	Pinocarveol	1138	0.67	0.97	0.30		1.14	1.54		1.48		0.31	0.69	0.46	2.13	0.95	2.42	1.96	**	0.152	**
**26**	**Camphor^d^**	1143	**10.23**	**9.40**	**6.80**	**8.17**	**9.93**	**11.72**	**12.02**	**8.16**	**19.39**	**14.37**	**19.93**	**15.69**	**17.49**	**13.64**	**18.59**	**14.00**	***	0.076**†**	***
27	p-Menth-4(8)-ene	1154	0.37	0.41		0.08	0.17	0.41	0.37		0.41		0.20			0.33			na	0.585	**
28	Pinocarvone	1159	0.87	0.55	0.13	0.28	0.59	0.43	0.82	0.81	1.44	0.89	1.17	1.09	0.91	0.53	1.18	1.16	***	0.076**†**	***
29	Borneol	1164	4.58	5.19	3.47	3.33	4.09	4.18	6.86	5.40	5.37	4.69	6.21	6.14	5.04	4.60	5.94	4.98	***	0.076**†**	**
**30**	**4-Terpineol^d^**	1177	4.36	4.57	5.30	3.32	**8.34**	**11.86**	2.87	1.78	2.94	1.79	2.70	2.38	2.36	**8.56**	1.55	1.63	***	0.146	***
31	p-Cymen-8-ol^e^	1184	0.57	0.76	0.30	0.25	0.46	1.23	1.00	1.01	1.08	0.75	0.71	0.59	0.65	1.16	0.52	0.32	***	0.304	***
32	1-α-Terpineol	1189	1.40	1.39	1.90	1.43	1.46	1.29	1.42	1.92	1.01	1.32	1.07	1.35	1.02	1.26	0.92	1.21	***	0.112	**
33	Myrtenal^e^	1193	1.56	1.60	0.20	0.98	1.81	1.46	2.40	1.62	3.16	1.79	2.62	2.30	2.08	1.58	2.69	2.42	***	0.072**†**	***
34	*trans*-Piperitol	1204				0.04		0.27								0.09			na	0.429	ns
35	Verbenone	1206	1.34	1.55	0.12	0.34	0.88	1.13	1.43	1.05	2.55	1.47	1.92	1.96	1.70	1.33	2.39	1.42	***	0.131	***
36	*trans*-Carveol	1216	0.58	0.73		0.21	0.33	0.47	1.43	1.22	1.36	0.80	0,86	0.91	0.74	0.65	0.81	0.85	***	0.064**†**	***
37	Thymyl methyl ether^e^	1228	0.70	0.20	1.21	0.24	0.36	0.25	0.77							0.14			**	0.159	***
38	Cuminal	1236	0.03	0.12	0.38	0.11		0.17	0.16		0.13		0.03			0.16			**	0.776	ns
39	Carvone	1240	0.26	0.24	0.05	0.08	0.08	0.19	0.52	0.46	0.49	0.30	0.33	0.40	0.26	0.25	0.30	0.37	***	0.061**†**	***
40	Linalyl acetate^e^	1253	0.68	0.67			0.19	0.25											na	0.051**†**	**
41	Bornyl acetate	1286	2.32	3.28	1.42	2.61	1.80	2.60	4.36	7.56	2.88	1.40	2.29	1.67	1.84	3.00	1.24	1.66	***	0.230	**
42	Cuminol^e^	1295	0.07	0.32		0.05	0.13	0.20	0.14	0.40		0.05				0.21		0.05	na	0.092**†**	**
43	Thymol	1302			0.95	0.20		0.05								0.07			na	0.127	ns
44	Carvacrol^e^	1312			2.55														na	0.423	***
45	p-Mentha-1,4-dien-7-ol^e^	1332	0.22	0.47			0.15	0.37	0.13							0.27			na	0.099**†**	**
**46**	**Terpenyl acetate^d, e^**	1351							**8.88**	**14.92**	3.22	2.20	0.43	0.22	1.72	1.36			na	0.051**†**	***
47	Carvacryl acetate	1372			0.15														na	0.430	ns
48	α-Copaene	1378	0.14	0.09			0.22	0.07		0.08									na	0.110	**
49	β-Bourbonene	1388	0.01	0.01			0.08	0.07		0.08									na	0.134	***
50	β-Elemene	1393	0.06		0.22	0.26				0.08									na	0.162	**
51	α-Gurjunene^e^	1412	0.64	0.38	1.37	1.97	0.24			0.24									na	0.099**†**	***
52	*trans*-Caryophyllene	1424	0.67	0.19	1.73	1.65	0.57	0.24	0.24	1.12	0.15	0.74	0.37	0.59	0.16	0.44	0.10	0.70	***	0.520	**
53	α-Humulene^e^	1450			0.07	0.04													na	0.051**†**	**
54	Aromadendrene	1466	0.37	0.24	0.83	0.93	0.10			0.29	0.13	0.40	0.15	0.22	0.14	0.14	0.12	0.53	***	0.277	**
55	Germacrene D	1486	0.58	0.21	0.25	0.17	0.62	0.17	0.10	0.33		0.13		0.08		0.15			*	0.095**†**	*
56	Alloaromadendrene^e^	1487	0.04	0.06	0.13	0.19													na	0.051**†**	**
57	Bicyclogermacrene	1493	1.03	0.54	0.93	1.24	0.16					0.94	0.11	0.25		0.16		0.48	***	0.240	**
58	Eremophilene	1500	0.15	0.12						0.18									na	0.155	*
59	γ-Cadinene^e^	1511	1.83	1.25	2.44	2.58	0.98	0.55				0.19		0.07		0.38		0.13	***	0.070**†**	***
60	δ-Cadinene	1518	0.14	0.12	0.72	0.51	0.13		0.13	0.40	0.14	1.29	0.29	0.38	0.19	0.27		1.04	**	0.446	**
61	Elemol^e^	1546	0.03	0.03	1.66		1.13	0.31	0.22	0.96	0.36		0.39	0.35		0.17			*	0.385	*
62	Palustrol^e^	1567	0.05	0.25	0.19	0.32		0.05											na	0.070**†**	**
63	1,6-Germacradien-5-ol	1574			1.15							0.49						0.21	na	0.593	*
64	Spathulenol^e^	1578	1.41	1.59	0.09	0.83	0.12	0.19	0.34	1.05	1.10	1.76	0.71	1.05	0.59	0.90	0.97	2.21	***	0.075**†**	***
65	Caryophyllene oxide^e^	1583	1.80	1.56	1.05	2.66	1.90	1.62	3.90	5.55	2.96	2.30	3.32	3.87	1.72	2.19	2.72	4.42	***	0.131	***
**66**	**Viridiflorol^d^**	1593	3.62	4.24	5.69	**11.49**	2.16	3.25	0.88	0.55						2.57		0.17	***	0.068**†**	***
67	Ledol	1616	0.45	0.59	0.78	1.15	0.15	0.34	0.18	0.30	0.22	0.37	0.18	0.26	0.11	0.44	0.13	0.40	**	0.234	*
68	γ-Eudesmol	1630	0.2		0.32		0.12	0.12	0.50	0.24			0.09	0.17		0.06			na	0.233	ns
69	α-Cadinol	1639	2.1	1.57	3.58	3.4	1.43	0.67	0.10	0.44	0.46	1.40	0.38	0.52	0.18	0.83		1.07	***	0.124	***
70	β-Eudesmol	1652	0.86	0.76	2.22	0.86	1.06	0.80	0.79	2.08	0.92	2.67	1.13	0.97	0.32	1.30	0.62	2.65	*	0.760	ns
71	t-Muurolol	1660	1.67	1.39	0.04	1.13	0.62	0.70		2.23		0.28		0.56		0.67		0.30	**	0.157	**

Total identified (%)			97.72	95.33	98.09	96.29	89.51	96.72	94.41	88.99	97.50	91.57	99.76	94.65	97.46	95.84	97.19	93.43			

Grouped components (%)																					
Monoterpene hydrocarbons			30.08	26.68	27.77	29.68	28.22	29.69	20.36	8.49	24.68	26.72	28.21	25.84	29.22	26.96	28.15	26.19			
Oxygenated monoterpenes			45.92	49.02	42.08	32.01	46.97	54.21	51.85	41.42	59.28	47.47	61.03	56.88	60.97	53.16	62.18	51.12			
Sesquiterpene hydrocarbons			5.66	3.21	8.69	9.54	3.10	1.10	0.47	2.8	0.42	3.69	0.92	1.59	0.49	1.54	0.22	2.88			
Oxygenated sesquiterpenes			12.19	11.98	16.77	21.84	8.69	8.05	6.91	13.4	6.02	9.27	6.20	7.75	2.92	9.13	5.11	11.43			
Others			3.87	4.44	2.78	3.22	2.53	3.67	14.82	22.88	7.10	4.42	3.40	2.59	3.86	5.05	1.53	1.81			

^a^The numbering refers to elution order of compounds from a HP-5MS column and their percentages were obtained by FID peak-area normalization. The percentage for each population represents the average calculated on *n *individuals (3 <*n *< 5). ^b^RI, retention indices calculated against C_8_-C_25 _n-alkanes mixture on the HP 5MS column. ^c^For the detailed description of the populations (1-8) locations, see Table 1 and Figure 1. ^d^Major compound in bold fond. ^e^Compounds with a statistically significant variation among populations and phenological stages. *P1*: *p*-values using Fisher test (one-way analysis of variance) applied for normally distributed variables and considering only population effect. Fisher test was not applicable (na) for non-Normal distributed variables. *P2*: *p*-values using non-parametric Friedman test (two-way analysis of variance). *P2 *is a global *p*-value (population and phenological stage effect) is considered significant (**†**) at *p *≤ 0.1. *P3*: *p*-values using non-parametric Kruskal-Wallis test (one-way analysis of variance) considering only population effect. *P1 *or *P3 *are extremely significant (***) at *p *≤ 0.001, highly significant (**) at 0.001 ≤ *p *≤ 0.01, significant (*) at 0.01 ≤ *p *≤ 0.05 and not significant (ns) at *p *> 0.05.

### Chemical variation according to population locations and phenological stages

Our results showed that Fisher test was not applicable for 21 essential oil compounds (non-Normal distributed variables) (*P1*, Table 2). The analysis of variance showed that the means for the majority (47 over 50) of the oil compounds differed significantly among populations (*P1*, Table 2) and not between phenological stages (*p *> 0.05). In fact, only 11 compounds (sabinene **6**, β-myrcene **8**, *trans*-β-ocimene **14**, *cis*-sabinene hydrate **16**, camphenilone **18**, p-cymen-8-ol **31**, thymyl methyl ether **37**, γ-cadinene **59**, elemol **61**, spathulenol **64 **and caryophyllene oxide **65**) differed significantly (*p *< 0.05) between vegetative and flowering stages (data not shown). According to Fisher test, 9 over 50 compounds differed significantly among populations and between phenological stages, which are: sabinene **6**, *cis*-sabinene hydrate **16**, camphenilone **18**, p-cymen-8-ol **31**, thymyl methyl ether **37**, γ-cadinene **59**, elemol **61**, spathulenol **64 **and caryophyllene oxide **65**.

Among the 21 essential oil compounds (non-Normal distributed variables) non-parametric statistical tests such as Friedman (two-way analysis of variance) and Kruskal-Wallis (one-way analysis of variance) tests were applied. The resulting *p*-values of Friedman test, which are global *p*-values reflecting the combined effect of population location and phenological stage, were found to be significant at *p *< 0.1 for 8 over 21 compounds (linalyl acetate **40**, cuminol **42**, p-mentha-1,4-dien-7-ol **45**, terpenyl acetate **46**, α-gurjunene **51**, α-humulene **53**, alloaromadendrene **56 **and palustrol **62**) (*P2*, Table 2). Moreover, according to the Kruskal- Wallis test, the analysis of variance showed that means of 17 over 21 compounds differed significantly at *p *< 0.05 among populations (*P3*, Table 2). Besides, according to the same test, only 5 over 21 compounds (*trans*-piperitol **34**, cuminol **42**, carvacrol **44**, α-gurjunene **51 **and γ-eudesmol **68**) differed significantly at *p *< 0.05 between vegetative and flowering stages (data not shown). Therefore, according to non-parametric statistical tests, 9 over 21 compounds (linalyl acetate **40**, cuminol **42**, carvacrol **44**, p-mentha-1,4-dien-7-ol **45**, terpenyl acetate **46**, α-gurjunene **51**, α-humulene **53**, alloaromadendrene **56 **and palustrol **62**) differed significantly among populations and between phenological stages.

By combining all tests, the amounts of only 18 over 71 compounds (sabinene **6**, *cis*-sabinene hydrate **16**, camphenilone **18**, p-cymen-8-ol **31**, thymyl methyl ether **37**, linalyl acetate **40**, cuminol **42**, carvacrol **44**, p-mentha-1,4-dien-7-ol **45**, terpenyl acetate **46**, α-gurjunene **51**, α- humulene **53**, alloaromadendrene **56**, γ-cadinene **59**, elemol **61**, palustrol **62**, spathulenol **64 **and caryophyllene oxide **65**) showed a statistically significant variation among population locations and phenological stages (Table 2). Furthermore, taking into account all the oil identified compounds, a general model (Wilks' Lambda, two way analysis) was applied. In fact, a significant effect of the population location was also observed at *p *< 0.001. Nevertheless, neither the phenological stage, nor the interaction between population location and phenological stage were found to be statistically significant on the chemical composition of *T. algeriensis *essential oil (*p *> 0.05) (data not shown).

Table 2 showed that α-pinene **3**, 1,8-cineole **13 **and camphor **26 **were the major compounds present in most populations belonging to different bioclimates. α-Pinene **3 **was highly (7.41-13.94%) represented in most populations. Percentages of 1,8-cineole **13 **ranged from 7.55% (population 2) to 22.07% (population 7). The amounts of camphor **26 **ranged from 6.8% (population 2) to 19.93% (population 6), whereas Ben ElHadj Ali et al. [6] showed that camphor characterized few populations from the semi-arid zone of Tunisia. In addition to the compounds already described, 4-terpineol **30 **mainly characterized the inferior arid zone population 3 (8.34-11.86%) (Table 2). In our recent work [3], 4-terpineol was also found at relatively high rate (7.36%) in a population from the inferior arid zone. Nevertheless, the amounts of 4-terpineol were very low (< 0.1%) in other described Tunisian populations from the same bioclimatic zone [6]. Our results also showed that *cis*-sabinene hydrate **16 **characterized only population 2 (9.86%) and population 3 (12.95%) from the inferior arid zone at the vegetative stage (Table 2). Recently, *cis*-sabinene hydrate was also found at relatively high rate (5.29%) in a population from the same region at the flowering stage [3]. However, this compound was not detected in Tunisian populations described by Ben ElHadj Ali et al. [6] and it was reported at low amounts (0.15-2.30%) in Algerian populations [4,5]. The amounts of terpenyl acetate **46 **were very low (< 3.22%), except for population 4 (inferior semi-arid bioclimate), which was distinguished by a high proportion of this constituent (8.88-14.92%) (Table 2). Interestingly, terpenyl acetate **46**, whose identification was confirmed by ^13^C-NMR spectroscopy, was described for the first time as a main compound in the essential oil of Tunisian *T. algeriensis*. Viridiflorol **66 **has the highest percentage in the inferior arid population 2 (5.69-11.49%) (Table 2). A similar result was previously described by Ben ElHadj Ali et al. [6] in populations from the same bioclimatic region. Nevertheless, this compound was not found in the essential oils of Algerian populations of *T. algeriensis *[5]. As can be seen in Table 2, in populations from the superior semi-arid bioclimate, borneol **29 **was detected with low amounts (4.60-6.21%) as compared to what has been obtained by Ben ElHadj Ali et al. [6] where this compound ranged between 18.4 and 24.3%. Thymol **43 **was absent in the most of populations (Table 2). By contrast, this compound was found to be highly represented (54.9%) in a population from the superior-arid zone of Tunisia [6] and the percentage of thymol in Algerian *T. algeriensis *ranged from 0.2 to 29.5% [5].

### Chemical clusters among populations

**Figure 2:**
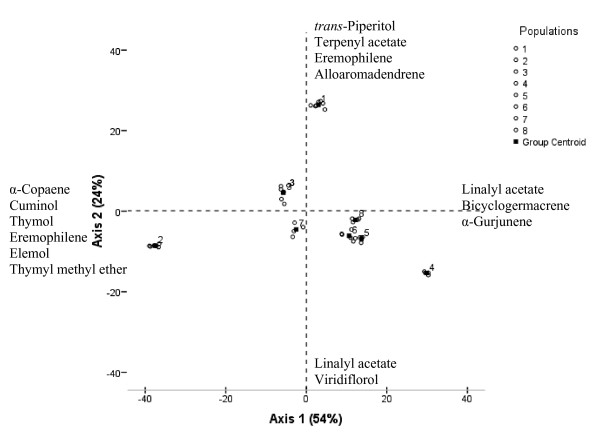
**Linear descriminant analysis (LDA) for the essential oil compounds of the 8 *T. algeriensis *populations**. Projection of the average contents of the essential oil compounds onto the first two principal axes (+ and - indicate positive and negative correlations with the axes, respectively). Coding numbers of populations' locations and for the detailed description of the bioclimatic zones: see Table 1.

To identify possible relationships between volatile compounds and geographical locations, linear discriminate analysis (LDA) was applied. The LDA, performed on average contents of all compounds for each population regardless the phenological stage, showed that the first two principal axes represented 77.90% of the total variation. The first axis (54% of the total variation) was mainly correlated with linalyl acetate **40**, α-gurjunene **51**, α-copaene **48**, cuminol **42**, thymol **43**, eremophilene **58**, elemol **61**, thymyl methyl ether **37 **and bicyclogermacrene **57**. The second axis represented 24% of the total variation, and linalyl acetate **40**, viridiflorol **66**, terpenyl acetate **46**, *trans*-piperitol **34**, eremophilene **58 **and alloaromadendrene **56 **were the main compounds contributing to its definition. The plot of the projection of the average values of all the compounds onto the first two principal axes, revealed a high chemical dispersion among populations (Figure 2). Furthermore, when using only the 18 compounds which show a statistically significant variation among populations and phenological stages (Table 2), the plot according to axes 1 and 2 (70.70% of the total variation) showed a similar chemical population groups (data not shown). Therefore, according to the linear discriminate analysis, four population groups in relation to the geographic location could be distinguished. The first, the second and the third group represented by populations 2, 4 and 1 respectively, situated at the periphery of the plot. Population 2 (inferior arid zone), situated at the negative sides of axes 1 and 2, constituted the first group. Population 4 (inferior semi-arid zone), situated at the positive side of axis 1 and at the negative side of axis 2, formed the second group. Population 1 from the inferior arid zone is situated at the positive sides of axes 1 and 2 formed the third group. The fourth group, situated in the centre of axis 1 and 2, is represented by the populations 3 from the inferior arid bioclimate and populations 5, 6, 7 and 8 from the superior semi-arid bioclimate.

**Figure 3:**
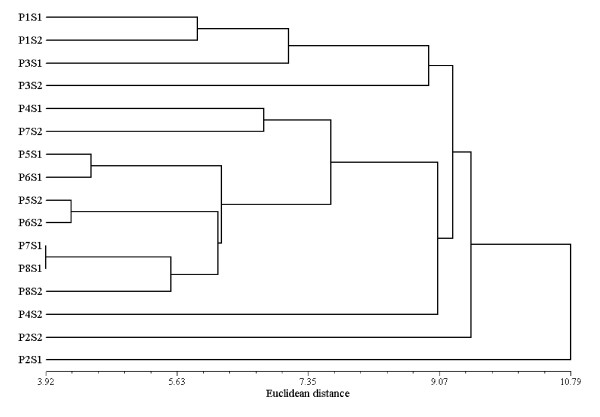
**Dendrogram obtained by cluster analysis based on Euclidean distance performed on the essential oil compounds of the 8 *T. algeriensis *populations during two phenological stages**. PiSj represent population i (i = 1-8) at the vegetative stage (S1) or at the flowering stage (S2). Coding numbers of populations' locations: see Table 1.

In addition to the linear discriminate analysis and to better characterize populations groups, cluster analysis (dendrogram) was applied to a matrix linking essential oil composition to sample location and phenological stages. In fact, the dendrogram generated from the Euclidean distances (Figure 3) performed on the essential oils compounds of *T. algeriensis *populations at each phenological stage, showed population groupings globally similar to those observed by the LDA clustering. In fact, the general structure of the dendrogram showed the existance of three main clusters. The first group included populations 1 and 3 from the inferior arid bioclimate which could be divided into two subgroups represented by population 1 and population 3, respectively. The dendrogram showed that population 3 had a greater affinity with the population 1. Moreover, LDA clustering also showed that the axis 2 divided populations into two major groups where population 3 trended with population 1 (Figure 2). The second group also contained two subgroups represented by populations (5, 6, 7S1 and 8) and populations (4 and 7S2), respectively, while the third cluster was represented by population 2.

Principal axes of Figure 2 showed that essentially minor compounds played an important role to distinguish between the chemical groups. However, conventional essential oil chemotypes were determined only on the basis of major compounds and therefore, five *T. algeriensis *chemotypes could be distinguished. The first group was represented by population 2 characterized by oils rich in α-pinene **3/**1,8-cineole **13/**camphor **26 **and it is distinguished from other populations by the presence of *cis*-sabinene hydrate **16 **at the vegetative stage and viridiflorol **66 **at the flowering stage as major compounds. By contrast to other populations, population 4 was characterized by the highest percentage of terpenyl acetate **46 **and other compounds (14.82-22.88) that did not belong to the monoterpenes and the sesquiterpenes (Table 2). Population 4 can be defined as 1,8-cineole **13/**camphor **26**/terpenyl acetate **46 **chemotype and formed the second group. Populations 1, 5, 6, 7/S1 and 8 can be defined as α-pinene **3/**1,8-cineole **13/**camphor **26 **chemotype and formed the third group. The forth group represented by populations 3 and 7 at the flowering stage (3/S2 and 7/S2) which corresponded to a chemotype rich in 1,8-cineole **13**/camphor **26**/4-terpineol **30**. The fifth group represented by population 3 at the vegetative stage (3/S1) presented an essential oil rich in α-pinene **3/**1,8-cineole **13/***cis-*sabinene hydrate **16/**camphor **26**/4-terpineol **30**.

In our study, Tunisian *T. algeriensis *showed a high chemical diversity among populations from the same region and bioclimate. In fact, populations 1, 2 and 3 from the inferior arid bioclimate and which were geographically close populations, clustered separately into different chemotypes. Nevertheless, the northern populations 5, 6, 7 and 8 from the superior semi-arid bioclimate and which were geographically near each other constituted an homogeneous group (Figure 2 and 3). Ben ElHadj Ali et al. [6] also showed a high chemical polymorphism among *T. algeriensis *populations. They showed that distribution of essential oil chemotypes was not always concordant with the bioclimatic zones and seemed rather to be linked with the geographic location and local selective forces acting on the chemotype diversity. In fact, local abiotic (topography, moisture, temperature and edaphic factors) and/or biotic selective factors (associated fauna and flora) act on loci terpene biosynthesis pathways and contribute to the emergence of different chemical profiles [7].

### Influence of population location and phenological stage on yield and metal chelating activity of essential oil

The essential oils extracted by hydrodistillation from the dried aerial parts of *T. algeriensis*, collected from diverse locations during the vegetative and the flowering stages, ranged from 1.03 to 3.66% (v/w) (Table 3). These yields were higher in South West of Tunisia (populations 1, 2 and 3 from the inferior arid bioclimate) than in North West of Tunisia (populations 5, 6, 7 and 8 from the superior semi-arid bioclimate) with a maximum obtained in the population 2. It was reported that climatic conditions, soil types of collected regions and different phases of the plant development induce high variations in essential oil yield and their compounds [8]. Besides, for populations 5 and 7 these yields significantly decreased (*p *< 0.01) from the vegetative to the flowering stage (Table 3). Similar results were previously obtained for *Malva aegyptiaca *[9] where the yields of volatiles decreased from vegetative stage, full-flowering plants to seed-bearing plants. Nevertheless, the essential oil yields were found to be higher during the flowering phase than in the vegetative stage of *Thymus capitatus *[10] or *Thymus caramanicus *[11].

**Table 3:** Yield and chelating activity of *T. algeriensis *essential oils during the vegetative and the flowering stages. Coding numbers of populations' locations: see Table 1

Populations	Vegetative stage		Flowering stage		Student test	
	Yield (ml/100 g)	IC_50 _(μg/ml)	Yield (ml/100 g)	IC_50 _(μg/ml)	Y	C
1	2.68 ± 0.58^b^	180 ± 15^d^	2.38 ± 0.23^b^	190 ± 17^bc^	ns	ns
2	3.66 ± 0.28^c^	140 ± 15^c^	3.66 ± 1.0^c^	130 ± 0.0^ab^	ns	ns
3	2.70 ± 0.30^b^	50 ± 5^a^	2.25 ± 0.01^b^	240 ± 40^c^	ns	*
4	1.70 ± 0^a^	86 ± 5^ab^	1.44 ± 0.19^a^	70 ± 20^a^	ns	ns
5	3.0 ± 0^b^	80 ± 5^a^	1.35 ± 0.10^a^	140 ± 60^ab^	**	ns
6	1.85 ± 0.22^a^	120 ± 45^bc^	1.68 ± 0.10^ab^	120 ± 80^ab^	ns	ns
7	1.88 ± 0^a^	76 ± 5^a^	1.03 ± 0.06^a^	68 ± 3^a^	**	ns
8	1.76 ± 0^a^	280 ± 40^e^	1.23 ± 0.44^a^	100 ± 45^a^	ns	**

Values represent mean ± standard deviation. Values followed by the same letter under the same row, are not significantly different (*p *> 0.05). For the same population, Student test was used to compare averages of essential oil yield (Y) and chelating activity (C) between phenological stages and is considered highly significant (**) at 0.001 ≤ *P *≤ 0.01, significant (*) at 0.01 ≤ *P *≤ 0.05 and not significant (ns) at *P *> 0.05.

Metal chelating activity was known as one of antioxidant mechanisms, since it reduced the concentration of the catalyzing transition metal in lipid peroxidation. Among the transition metals, Fe^2+ ^ion was known as the most important lipid oxidation prooxidant due to its high reactivity [12]. Chelating activity is presented by IC_50 _value, defined as the concentration of the essential oil needed to chelate 50% of Fe^2+ ^present in the test solution and calculated from the graph of chelating percentage against extract concentration. Lower IC_50 _value reflected better chelating activity. Essential oils of *T. algeriensis *collected from diverse locations during the vegetative and the flowering stages were subjected to screening for their chelating activities (Table 3). Our results showed that statistically significant differences of chelating activity were mainly observed when they were compared by the population location criteria. In fact, the variation of phenological stages did not have a statistically significant effect on the oil chelating activity for the most of populations (Table 3). Chelating activity was found to be very interesting (from 68 to 86 μg/ml) for populations 4 and 7 and which were comparable to the chemical EDTA (IC_50 _value = 40 μg/ml). Nevertheless, in the work of Bounatirou et al. [10], the antioxidant activity (DPPH assay) of essential oils obtained from the aerial parts of *T. capitatus *varied by the period of vegetation (vegetative, flowering or post-flowering) but no major differences were found between the antioxidant activity of the oils collected at different locations.

## Conclusions

Analysis by GC/FID, GC/MS and ^13^C-NMR of Tunisian *T. algeriensis *essential oils allowed the identification of 71 compounds. The major compounds at the species level were α-pinene (7.41-13.94%), 1,8-cineole (7.55-22.07%), camphor (6.8-19.93%), 4-terpineol (1.55-11.86%), *cis-*sabinene hydrate (0.10-12.95%), terpenyl acetate (0-14.92%) and viridiflorol (0-11.49%). A high variation among populations was revealed for the majority of oil compounds. Nevertheless, neither the phenological stage, nor the interaction between population location and phenological stage were found to be statistically significant on the chemical composition of *T. algeriensis *essential oil. The spatial distribution of the populations was not concordant with the bioclimatic zones and seemed rather to be liked to local selective forces acting on the chemotype diversity. It is worthy to note that in the linear discriminant analysis, essentially minor compounds play an important role to distinguish between the chemical groups. Based on major compounds, the populations were represented by (α-pinene/1,8-cineole/*cis*-sabinene hydrate/camphor/viridiflorol), (1,8-cineole/camphor/terpenylacetate), (α-pinene/1,8-cineole/camphor), (1,8-cineole/camphor/4-terpineol) and (α-pinene/1,8-cineole/*cis*-sabinene hydrate/camphor/4-terpineol) chemotypes. The metal chelating activity of the essential oils was assessed and compared to synthetic EDTA. A variation of metal chelating activity of the oil was revealed according to population locations rather than to bioclimates or phenological stages. These results can be used to investigate the geographical location and the harvesting time of this plant for relevant industries.

## Methods

### Populations analyzed and sampling

The 8 populations of *T. algeriensis *collected from different bioclimatic and geographical zones and reported in Table 1 and Figure 1 were analyzed separately. A number of three to five individuals from each population were sampled over the entire population area at the vegetative (December 2009) and at the flowering (April 2010) stages. The distance between individuals exceeded 20 m, to avoid collection from close parents. The harvested samples size does not exceed 20 cm. After that, the fresh vegetable matter was first weighted and then dried on the shadow, until constancy of the weight (20 days). Separated from stems, aerials parts were subjected for essential oil extraction.

### Essential oil extraction

The dry matter was submitted to hydrodistillation for 4 h, using a Clevenger-type apparatus. Each essential oil was dried over anhydrous sodium sulphate and stored in sealed vials protected from light at -20°C until analysis.

### Essential oil analyses

#### Gas chromatography (GC)

A Hewlett-Packard 5890 series II gas chromatograph equipped with HP-5MS capillary column 30 m × 0.25 mm i.d., film thickness 0.25 μm; Hewlett-Packard) and connected to a flame ionization detector (FID) was used. The column temperature was programmed at 50°C for 1 min, then 7°C/min to 250°C, and then left at 250°C for 5 min. The injection port temperature was 240°C and that of the detector 250°C (split ratio: 1/60). The carrier gas was helium (99.995% purity) with a flow rate of 1.2 ml/min and the analysed sample volume was 2 μl. Percentages of the constituents were calculated by electronic integration of FID peak areas, without the use of response factor correction. Mean percentage of compounds in *T. algeriensis *essential oil represents the average calculated on three to five individuals. Retention indices (RI) were calculated for separate compounds relative to (C_8_-C_25_) n-alkanes mixture (Aldrich Library of Chemicals Standards) [13].

#### Gas chromatography/mass spectrometry (GC/MS)

The isolated volatile compounds were analysed by GC/MS, using a Hewlett-Packard 5890 series II gas chromatograph. The fused HP-5MS capillary column (the same as that used in the GC analysis) was coupled to a HP 5972A masse-selective detector (Hewlett-Packard, Palo Alto, CA, USA). The oven temperature was programmed at 50°C for 1 min, then 7°C/min to 250°C, and then left at 250°C for 5 min. The injection port temperature was 250°C and that of the detector was 280°C (split ratio: 1/100). The carrier gas was helium (99.995% purity) with a flow rate of 1.2 ml/min and the analysed sample volume was 2 μl. The mass spectrometer conditions were as follow: ionization voltage, 70 eV; ion source temperature, 150°C; electron ionization mass spectra were acquired over the mass range 50-550 m/z.

#### Volatile compounds identification

The essential oil compounds of *T. algeriensis *were identified by comparing the mass spectra data with spectra available from the Wiley 275 mass spectra libraries (software, D.03.00). Further identification confirmations were made referring to retention indices (RI) data generated from a series of known standards of n-alkanes mixture (C_8_-C_25_) [13] and to those previously reported in the literature [12,14-22].

#### ^13^C-NMR analysis

NMR spectra were recorded on a Bruker AVANCE 400 Fourier Transform spectrometer operating at 100.13 MHz for ^13^C-NMR, equipped with a 5 mm probe, in CDCl_3_, with all shifts referred to internal TMS. ^13^C-NMR spectra of the oil samples were recorded with the following parameters: pulse width = 4 μs (flip angle 45°); acquisition time = 2.7 s for 128 K data table with a spectral width of 25 000 Hz (250 ppm); CPD mode decoupling; digital resolution = 0.183 Hz/pt. The number of accumulated scans was 2000-3000 for each sample depending of the available amount of oil (when available, 40 mg of oil in 0.5 ml of CDCl_3_). Identification of some compounds such as terpenyl acetate was assessed by the method developed and computerized in the laboratory of the team *"Chimie et Biomasse"*, using home-made software, by comparison with spectral data of reference compounds compiled in a laboratory-built library [23].

#### Metal (Fe^2+^) chelating activity

Chelating activity of the essential oils was assessed by the ferrozine assay as described by Dinis et al. [24]. Ferrozine can quantitatively form complexes with Fe^2+^. In the presence of other chelating agents, the complex formation is disrupted with the result that the red color of the complex is decreased. Therefore, measurement of the rate of color reduction allows estimation of the chelating activity of the coexisting chelator. To 0.5 ml of essential oil solution prepared in methanol, 1.6 ml of deionised water and 0.05 ml of FeCl_2 _4H_2_O solution (2 mM) were added and left for incubation at room temperature for 5 min. Then, the reaction was initiated by adding 0.1 ml of ferrozine (5 mM), shaken vigorously and left standing at room temperature for 10 min. Absorbance of the solution was then measured at 562 nm. The chelating antioxidant activity for Fe^2+ ^was calculated according to the following formula:

$$\text{Metal chelating rate (\% )}=\left[\frac{\text{Ac - As}}{\text{Ac}}\right]\times 100$$

where Ac is the absorbance of the control reaction and As is the absorbance of the tested sample. Essential oil concentration (μg/ml) corresponding to 50% ferrous iron chelating (IC_50_) was calculated from the graph plotting Fe^2+ ^chelating activity against oil concentration. EDTA was used as a positive control and all determinations were carried out in triplicate.

#### Statistical analyses

The distribution of the 71 compounds identified from the essential oil was checked by a descriptive statistical analysis using the SPSS software for Windows™ (version 17, SPSS Inc., Chicago, IL, USA). The percentages of compounds were transformed using the arcsine transformation and all classic ones in order to improve the distribution property. However, these transformations did not yield satisfactory results. To assess the variation of the percentages of compounds (having Normal distribution) among the populations and/or phenological stages, a two-ways ANalysis Of VAriance (ANOVA) was performed using the SPSS software. For compounds having skewed distributions we have performed a non parametric two-way analysis of variance (Friedman test) implemented in the statistical programming environment R version 2.7.1 (R Development Core Team R: A Language and Environment for Statistical Computing, Vienna, Austria; 2008). We also computed Kruskal-Wallis test (SPSS) to compare stages and to test difference between populations. Statistical significance was assumed at *p *< 0.05. The chemical population structure and the relationship among populations were determined by Linear Discriminate analysis (LDA) performed on the percentages of all identified compounds for all populations using the SPSS program. The divergence between populations was also estimated by the Euclidean distances calculated among population pairs using the Numerical Taxonomy and Multivariate Analysis System (NTSys) software version 2.1 [25]. A dendrogram representing all populations and phenological stages was constructed using the Euclidian distance calculated among populations/phenological stages pairs and the Unweighted Pair Group Method with Arithmetic Averaging (UPGMA) algorithm [26]. The resulting tree was plotted using the treeview software version 1.6.6 [27]. Duncan's multiple range test (*p *< 0.05) and Student test (*p *< 0.05) were used to compare averages of essential oil yield and chelating activity among populations and phenological stages, respectively.

## Competing interests

The authors declare that they have no competing interests.

## Authors' contributions

SZ (chemist) carried out chemical composition analysis of essential oils. IA and AR (statisticians) contribute to the statistical analyses. NF and NZ (biochemists) realized the essential oil extraction, metal chelating activity and the redaction of the manuscript. Authors read and approved the final manuscript.
